# Like dissolves like: A first-principles theory for predicting liquid miscibility and mixture dielectric constant

**DOI:** 10.1126/sciadv.abe7275

**Published:** 2021-02-12

**Authors:** Bilin Zhuang, Gabriele Ramanauskaite, Zhao Yuan Koa, Zhen-Gang Wang

**Affiliations:** 1 Division of Chemistry and Chemical Engineering, California Institute of Technology, Pasadena, CA 91125, USA.; 2 Yale-NUS College, Singapore 138527, Singapore.; 3 Institute of High Performance Computing, Singapore 138632, Singapore.

## Abstract

Liquid mixtures are ubiquitous. Miscibility and dielectric constant are fundamental properties that govern the applications of liquid mixtures. However, despite their importance, miscibility is usually predicted qualitatively based on the vaguely defined polarity of the liquids, and the dielectric constant of the mixture is modeled by introducing mixing rules. Here, we develop a first-principles theory for polar liquid mixtures using a statistical field approach, without resorting to mixing rules. With this theory, we obtain simple expressions for the mixture’s dielectric constant and free energy of mixing. The dielectric constant predicted by this theory agrees well with measured data for simple binary mixtures. On the basis of the derived free energy of mixing, we can construct a miscibility map in the parameter space of the dielectric constant and molar volume for each liquid. The predicted miscibility shows remarkable agreement with known data, thus providing a quantitative basis for the empirical “like-dissolves-like” rule.

## Teaser

The empirical “like-dissolves-like” rule for liquid miscibility can be quantified.

## INTRODUCTION

Liquid mixtures are ubiquitous. Dielectric constant and miscibility are two fundamental properties that govern the various applications of liquid mixtures. Yet, our understanding of these properties is still largely based on experience and experimentation. To determine whether two liquids mix well with each together, we mostly rely on the empirical “like-dissolves-like” rule in terms of the polarity of the two liquids. However, the definition of polarity remains vague—a “polarity index” has been defined on the basis of a solvent’s interaction with ethanol, dioxane, and nitromethane (*1*), but this definition is rather arbitrary and has not been widely used in subsequent literature. Even with a definition at hand, it is difficult to know quantitatively how “like” two liquids need to be in order to be miscible. For the dielectric constant ε of a binary liquid mixture, various expressions in the form of some types of averages have been proposed; some typical examples are (*2*–*5*)

$$\varepsilon =x_{A}\varepsilon_{A}+x_{B}\varepsilon_{B}$$ (1)

$$\varepsilon =\phi_{A}\varepsilon_{A}+\phi_{B}\varepsilon_{B}$$ (2)

$$\frac{1}{\varepsilon }=\frac{\phi_{A}}{\varepsilon_{A}}+\frac{\phi_{B}}{\varepsilon_{B}}$$ (3)

$$\varepsilon^{\frac{1}{3}}=\phi_{A}\varepsilon_{A}^{\frac{1}{3}}+\phi_{B}\varepsilon_{B}^{\frac{1}{3}}$$ (4)

$$\varepsilon =\varepsilon_{A}^{\phi_{A}}\varepsilon_{B}^{\phi_{B}}$$ (5)

where ε*_S_* denotes the dielectric constant of the pure liquid *S*, and *x_S_* and ϕ*_S_* are the mole fraction and the volume fraction of species *S*, respectively.

Theories on liquid mixtures are generally developed by introducing mixing rules. Equations 1 to 5 are examples of mixing rules for a mixture’s dielectric constant. Mixing rules are assumptions that are often introduced for macroscopic quantities such as the dielectric constant, but they have also been introduced for molecular quantities or interactions. For example, in the regular solution theory (also called as the van Laar–Scatchard-Hildebrand theory), which is the basis of the widely used solubility parameter, the major assumption is that the dispersion force between two species is the geometric mean of the dispersion forces in the pure substances, i.e., (*6*–*8*)

$$c_{\mathrm{AB}}=\sqrt{c_{\mathrm{AA}}c_{\mathrm{BB}}}$$ (6)

where *c*_*SS*^′^_ is the per-volume interaction energy between molecules of species *S* and *S*^′^. On the basis of this assumption, the free energy of mixing is written as

$$\beta \Delta f_{\text{mix}}=\beta \phi_{A}\phi_{B}{\left(\delta_{A}-\delta_{B}\right)}^{2}+\frac{\phi_{A}}{v_{A}}\text{ln}\;x_{A}+\frac{\phi_{B}}{v_{B}}\text{ln}\;x_{B}$$ (7)

where δ*_S_* is the solubility parameter of species *S*, defined as $\delta_{S}=\sqrt{\Delta u_{S}^{\text{vap}}}$, with $\Delta u_{S}^{\text{vap}}$ being the energy of vaporization per volume, β = 1/(*k*_B_*T*) is the inverse temperature scaled by the Boltzmann constant *k*_B_, and *v_S_* is the volume of a molecule of species *S*. The difference in solubility parameters is now a widely used measure for determining the miscibility between two liquids (*9*, *10*). However, despite its popularity, miscibility predictions based on Eq. 7 can often be quite a bit off, as will be shown later.

In this work, we develop a molecular-based theory for liquid mixtures of dipole molecules using a field-theoretic approach. A key effect in liquids of dipole molecules is the reaction field—the polarization in the surrounding medium induced by a tagged dipole (*11*). Because of this effect, a mean-field approach is insufficient to capture the dielectric properties of a polar liquid (*12*, *13*). We have recently shown that a nonperturbative treatment based on a renormalized Gaussian fluctuation theory (*12*) can naturally account for the reaction field effects and yield good predictions for the dielectric constant for a single-component fluid. This work generalizes that theory to liquid mixtures, with the goal to (i) predict the dielectric constant for the mixture and (ii) predict the miscibility between any two liquids based on their dielectric constants and molar volumes. We note that Fredrickson and coworkers (*13*–*16*) have published a series of papers that treat the polarization effects in liquids and liquid mixtures using a field-theoretic approach. Their discussions of liquid miscibility focused on nonpolar liquids (i.e., without permanent dipoles) and the comparisons to experimental data involved the use of adjustable parameters. Furthermore, their work used a bare one-loop expansion, which we have shown to give less accurate predictions on the dielectric constant of polar liquids than our renormalized Gaussian fluctuation theory (*12*). Instead of the one-loop expansion, we use a variational method to account for the reaction field effects. This results in a systematic treatment of dipole-dipole interactions in a liquid mixture and provides a theory that accurately describes liquid miscibility and mixture dielectric constant.

## RESULTS AND DISCUSSION

### Mixture dielectric constant

**Fig. 1:**
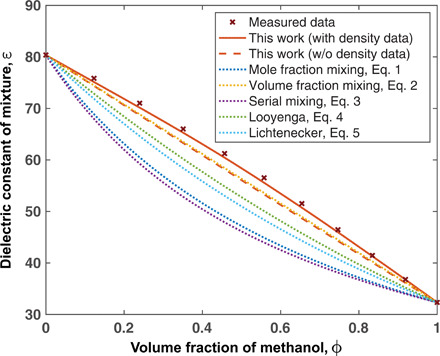
Dielectric constant of water-methanol mixture. ϕ denotes the volume fraction of methanol in the mixture. The predictions by the theory in this work with and without mixture density data are compared with the measured data (*36*) and with the predictions by the dielectric mixing rules (Eqs. 1 to 5).

For isotropic liquids, our theory results in the following simple expression for the dielectric constant of a mixture

$$\frac{\left(\varepsilon -1)(2\varepsilon +1\right)}{\varepsilon }=3y(\frac{2y^{2}+3y+9}{{\left(y+3\right)}^{2}})$$ (8)

where $y=\sum_{S}\frac{\beta {\bar{\mu }}_{S}^{2}\rho_{S}}{3\varepsilon_{0}}$, with ρ*_S_* being the number density, ${\bar{\mu }}_{S}$ the effective dipole moment of species *S* in the mixture, and ε_0_ the permittivity of vacuum. *y* is a dimensionless parameter characterizing the strength of dipolar interactions. Equation 8 is valid when there is no spatial inhomogeneity caused by the applied electric field. With Eq. 8, we can obtain the dielectric constant of the mixture using the dielectric constants of the pure components, without any fitting parameters or invoking ad hoc mixing rule: Given the dielectric constants ε*_S_* of the pure liquids, we obtain the effective dipole moments ${\bar{\mu }}_{S}$ by applying Eq. 8 to each pure liquid. Then, with data on the density of the mixture and the mixture composition, ρ*_S_* for each solvent in the mixture can be computed. Applying Eq. 8 to the mixture using ${\bar{\mu }}_{S}$ and ρ*_S_* then yields the dielectric constant of the mixture. Using water-methanol mixture as an example, we compare the prediction of our theory (shown as the red solid line) with measured data (shown as crosses) in Fig. 1. We see that the prediction by our theory agrees very well with the measured data.

Often, the density of the liquid mixture is not available and we have to predict the mixture’s dielectric constant on the basis of pure-liquid properties. To do so, we assume that there is no volume change upon mixing by writing ρ*_S_* = ϕ*_S_*/*v_S_*, where ϕ*_S_* is the (nominal) volume fraction of species S, and *v_S_* is the volume per molecule for the pure liquid S, given by the molar volume divided by Avogadro’s number. This leads to $y=\sum_{S}\beta {\bar{\mu }}_{S}^{2}\phi_{S}/(3\varepsilon_{0}v_{S})$. Under this assumption, the prediction of our theory is plotted with the red dashed line in Fig. 1 and compared to the dielectric mixing rules given by Eqs. 1 to 5. As can be seen, the prediction of our theory without knowledge of the mixture density is comparable to the volume-fraction mixing rule and better than all other mixing rules. We also observe that the slight deviation of our theory from the volume-fraction mixing rule is negative. To predict a positive deviation from the volume-fraction mixing rule, we need a negative volume change upon mixing so that the dipole moments of the species interact more strongly in the mixture.

**Fig. 2:**
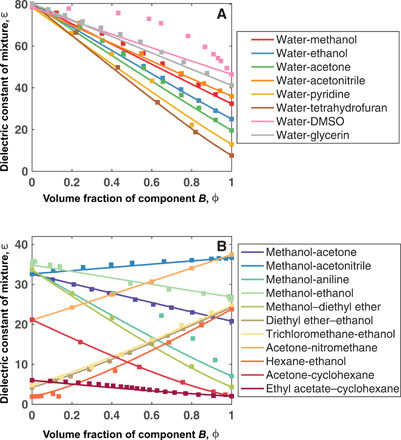
Dielectric constants of binary mixtures. (**A**) aqueous mixtures (**B**) nonaqeuous mixtures. The mixtures are labeled as *A-B*, with *A* and *B* representing the names of the solvents. ε is the dielectric constant of the mixture, and ϕ is the volume fraction of component *B*. The solid line is the dielectric constant predicted by our theory and the squared points represent measured data (*36*).

Under the assumption of no volume change upon mixing, we have found that the mixture dielectric constant of many liquids can be quite accurately described by our theory and follows approximately the volume-fraction mixing rule, as shown in Fig. 2. For cases where the mixture dielectric constant deviates slightly from the volume-fraction mixing rule, such as hexane–ethanol and acetone–cyclohexane mixtures, our theory provides a better prediction than the volume-fraction mixing rule. In rare cases, such as in the water–dimethyl sulfoxide (DMSO) mixture, the mixture dielectric constant shows substantial positive deviation from the volume-fraction mixing rule. This strong deviation suggests that the mixture is strongly nonideal that we must take into account additional correlations beyond the level of theory in this work. In the case of the water–DMSO mixture, it has been shown that water and DMSO form stronger hydrogen bonds than the hydrogen bonds in pure water (*17*).

**Fig. 3:**
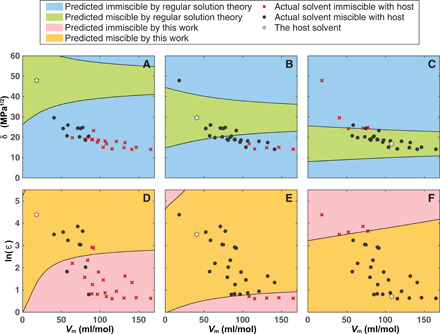
Miscibility maps. Results predicted by the regular solution theory (**A** to **C**) and by the statistical field theory in this work (**D** to **F**), compared to the measured data (*37*). The miscibility maps are for three host solvents: water (A and D), methanol (B and E), and cyclohexane (C and F). For the regular solution theory, the parameters involved are the solubility parameter δ and the molar volume *V*_m_. For the statistical field theory in this work, the parameters involved are the dielectric constant ε and the molar volume *V*_m_. The parameters for the host solvents are indicated by a white star in the respective plots. The scattered points indicate the miscibility of actual solvents with the respective host solvents. The data for computation are listed in the Supplementary Materials.

We note that our theory makes good predictions even for liquid mixtures including nonpolar substances such as hexane and cyclohexane, as shown in Fig. 2, even though the theory is developed for molecules with permanent dipole moments. This suggests that, for the purpose of predicting dielectric constant of the liquid mixture and liquid miscibility (see Fig. 3F), one can approximate induced-dipole interactions using an effective permanent dipole moment determined by the dielectric constant.

### Liquid miscibility

Assuming no change in volume upon mixing, our theory results in the following free energy of mixing for a homogeneous mixture

$$\begin{array}{cc} \beta \Delta f_{\text{mix}}= & \frac{1}{2}(\sum_{S}\frac{\phi_{S}y_{S}}{v_{S}})\frac{\text{ln}\;(1+\sum_{S}\phi_{S}y_{S})}{\sum_{S}\phi_{S}y_{S}}\\ & -\frac{1}{2}\sum_{S}(\frac{\phi_{S}}{v_{S}}\text{ln}\;(1+y_{S}))+\sum_{S}\frac{\phi_{S}}{v_{S}}\text{ln}\;\phi_{S} \end{array}$$ (9)

where *y_S_* is the value of *y* for the pure liquid *S*.

Since *y_S_* can be mapped to the dielectric constant ε*_S_* of the pure liquid *S* through Eq. 8 and molecular volume *v_S_* is simply the molar volume divided by Avogadro’s number, Eq. 9 can be used to predict the liquid miscibility based on the dielectric constant and molar volume. For two liquids to be completely miscible, Δ*f*_mix_ must be convex over all compositions. We can use this condition to generate a miscibility map for a given host liquid with other liquids. In Fig. 3 (D to F), we show the miscibility maps for water, methanol, and cyclohexane as host liquids. Liquids whose parameter values fall within the yellow region are completely miscible with the host liquid, while those that fall within the red region are immiscible at least for some compositions. We find remarkable agreement between our theoretical prediction and actual miscibility for these liquids. For comparison, we include miscibility maps for the same sets of liquids predicted by the regular solution theory (Fig. 3, A to C). We see that the predictions from the regular solution theory are quite a bit off for many liquids, especially with water and methanol as the host liquids. Just as for the dielectric constant, our theory makes good prediction for the miscibility involving the nonpolar cyclohexane (Fig. 3F), reaffirming the efficacy of treating induced-dipole interactions using an effective permanent dipole moment. The only clear discrepancies among this set of liquids are the five data points for water (the three black dots in the red region and the two red crosses in the yellow region in Fig. 3D): acetic acid, tetrahydrofuran, and 1,4-dioxane are miscible with water but our theory predicts them to be immiscible; 2-butanone and 1-butanol are immiscible with water but our theory predicts them to be miscible. These anomalies are likely due to molecule-specific hydrogen bonding effects that our theory does not take into account. For example, acetic acid is known to form dimers in the pure liquid, which break up upon solvation in water, so the use of Eq. 8 on the pure acetic acid underestimates the dipole moment of the acetic acid molecule (*18*, *19*). Given the complexity in hydrogen bonding in the aqueous environment, some discrepancies between the predictions from our theory and the experimental data are understandable.

The miscibility between two liquids is determined by the mismatch in both the dielectric constant and the molar volume. For two liquids *A* and *B* of equal molar volume, the free energy of mixing can be written in the form $\beta \Delta f_{\text{mix}}\approx \beta \phi_{A}\phi_{B}\eta {\left(y_{A}-y_{B}\right)}^{2}+\sum_{S}\frac{\phi_{S}}{v_{S}}\text{ln}\;\phi_{S}$ when it is expanded to the second order in *y_A_* − *y_B_*, with η being a constant. Thus, at the lowest order, the energy change due to mixing is consistent with that of the regular solution theory as presented in Eq. 7. The better prediction given by the free energy in this work (Eq. 9) is due to consideration of the molecular volume difference and the higher-order terms that result from the variational treatment.

In conclusion, using field-theoretic variational methods, we have formulated a first-principles molecular-based theory for dipole-dipole interactions in liquid mixtures. Without invoking any mixing rules as in many simple theories for liquid mixtures, our theory yields simple expressions for the dielectric constant of a mixture and free energy of mixing; predictions from our theory are in good agreement with the experimental data. In particular, for miscibility between liquids, we have shown that our theory makes accurate predictions based on the pure-liquid dielectric constants and molar volumes, thus providing a quantification for the well-known like-dissolves-like rule.

Liquid miscibility is a central consideration in many fields of science and technology, including separation/extraction (*20*, *21*), advanced materials formulation (*22*, *23*), food (*24*) and pharmaceutical (*25*, *26*) formulations, environment and sustainability (*27*, *28*), and even outer-space planet formation (*29*). Often, multicomponent mixtures are involved. The sheer number of different kinds and compositions of the mixtures makes it impossible to perform exhaustive experiments or simulations. A simple predictive theory is necessary to explore the many possibilities offered by mixtures. Our theory is a step in this effort.

## METHODS

### Model

We consider a liquid mixture at uniform density in an applied field **E**_0_(**r**). **E**_0_(**r**) will eventually be taken to be spatially uniform. For each species *S* in the liquid, there are *N_S_* molecules. The molecules are modeled as nonpolarizable, each having a permanent dipole moment ${\bar{\mu }}_{S}$ and volume *v_S_*. The microscopic state of the fluid can be specified by the set of positions {r_*S*,*i*_} and the dipole vectors {**μ**_*S*,*i*_} of all molecules, where the subscripts *S*,*i* refer to the *i*th molecule of type *S*.

To describe the electrostatic energy of the fluid mixture, we first introduce a microscopic polarization $\hat{P}(r)$, given by

$$\hat{P}(r)=\sum_{S}\sum_{i=1}^{N_{S}}\mu_{S,i}h_{S}(r-r_{S,i})$$ (10)

where *h_S_*(**r** − **r**_*S*,*i*_) is a function describing the local spread of the molecular polarization around the center of mass of the molecule. This function is defined in the same spirit as the local molecular charge distribution in (*30*). If we model the molecules as point dipoles, then *h_S_*(**r** − **r**_*S*,*i*_) = δ(**r** − **r**_*S*,*i*_). However, to render a finite self-energy for the dipoles, we allow a finite spread in the distribution. Mathematically, the only requirement for *h_S_* is that its integral in space is equal to 1, i.e., ∫*d***r** *h_S_*(**r** − **r**_*S*,*i*_) = 1. In this work, to keep the mathematics simple while describing the physics sufficiently, we assume a uniform molecular polarization in a sphere of volume $v_{S}^{\prime }$ around the center of the molecule, i.e.,

$$h_{S}(r-r_{S,i})=\{\begin{array}{cc} \frac{1}{v_{S}^{\prime }} & \text{if}\;|r-r_{S,i}|<{\left(\frac{3v_{S}^{\prime }}{4\pi }\right)}^{\frac{1}{3}}\\ 0 & \text{otherwise} \end{array}$$ (11)

The “dipole volume” $v_{S}^{\prime }$ can be considered an adjustable parameter to be obtained by fitting the pure-component dielectric constant. However, to make a priori predictions free of fitting parameters, we take this dipole volume to be the same as the physical volume by setting $v_{S}^{\prime }=v_{S}$. An alternative form for *h_S_* that describes the molecular polarization as a Gaussian distribution around the center of mass is discussed in the Supplementary Materials.

In terms of the polarization $\hat{P}(r)$, the electrostatic energy of the fluid is

$$U=\frac{1}{2}\int dr\int dr\prime \;\hat{P}(r)T(r-r\prime )\hat{P}(r\prime )-\int dr\;\hat{P}(r)\cdot E_{0}(r)$$ (12)

where **T**(**r**) = −∇∇(1/4πε_0_∣**r**∣) is the dipole-dipole interaction tensor. A detailed discussion of the relevant mathematical properties of **T**(**r**) has been presented in our earlier work in (*12*). Particularly useful is the Fourier transform of **T**(**r**) given by $\tilde{T}(k)=kk/\varepsilon_{0}k^{2}$. Here, we use a tilde above a quantity to denote the Fourier transform $\tilde{f}(k)=\int dr\;f(r)e^{-ik\cdot r}$.

We consider a grand canonical ensemble of a fluid mixture under chemical potential μ*_S_* for each species *S* at temperature *T* and volume *V*. The grand partition function of the system is

$$\Xi =\overset{\infty }{\sum_{N_{A}=0}}\ldots \overset{\infty }{\sum_{N_{B}=0}}\frac{e^{{\beta \mu }_{A}N_{A}}}{N_{A}!}\ldots \frac{e^{{\beta \mu }_{B}N_{B}}}{N_{B}!}Z$$ (13)

where *A* and *B* are representative labels of the solvent species and the “…” means similar summations and factors for other species need to be included if there are more than two species in the mixture. *Z* is the canonical partition function given by

$$Z=\prod_{S}\prod_{i=1}^{N_{S}}\int \frac{dr_{S,i}}{\Lambda_{S}^{3}}\int \frac{d\Omega_{S,i}}{4\pi }e^{-\beta U}$$ (14)

In Eq. 14, Λ*_S_* is the thermal de Broglie wavelength of species *S*, and the integral over the solid angle Ω_*S*,*i*_ accounts for the orientational degrees of freedom of the dipoles (since their magnitudes are fixed). It should be understood that in addition to the electrostatic interactions, there are also short-ranged excluded volume repulsions between molecules. Such interactions could be modeled, for example, by a local incompressibility constraint. However, for a homogeneous mixture with uniform density, such a constraint amounts to a simple shift in the chemical potentials under a mean-field treatment of the excluded volume effects; this is explicitly demonstrated in section S2. To avoid undue amount of mathematical details in the theory, here, we consider the effect of short-ranged excluded volume through the shifted chemical potentials, which can be determined by the liquid densities.

### Statistical field theory

The configurational integral in the partition function is intractable due to the pairwise dipole-dipole interactions in the liquid. To move forward, we decouple these pairwise interactions using the Faddeev-Popov method (*31*–*33*). This method allows us to transform the pairwise interaction into the interaction between polarization and a fluctuating field. By this method, we arrive at the formally exact field-based grand partition function

$$\Xi =\int DP\int DG\;e^{-L[P,G]}$$ (15)

where the effective field-theoretic action *L* is

$$\begin{array}{cc} L[P,G]= & \frac{1}{2}\int dr\int dr\prime P(r)T(r-r\prime )P(r\prime )\\ & +i\int drP(r)\cdot G(r)-\int drP(r)\cdot E_{0}(r)\\ & -\underset{S}{\Sigma }(\lambda_{S}\int dr\int d\Omega_{S}e^{i\int dr\prime h_{S}(r\prime -r)\mu_{S}\cdot G(r\prime )}) \end{array}$$ (16)

In the above expression, we have defined T=βT and $E_{0}=\beta E_{0}$ to simplify notation. $\lambda_{S}=e^{\beta \mu_{S}}/(4\pi \Lambda_{S}^{3})$ is the scaled fugacity of species *S*. **P** and *i***G** are, respectively, the fluctuating polarization and the conjugate fluctuating electric field, both of which are integrated over in Eq. 15.

### The variational approach

Through the field-theoretic transformation, evaluation of the partition function has been recast as integrals over fluctuating field variables. However, the last term in Eq. 16 that describes the interactions between single dipoles with the fluctuating field *i***G** makes the overall form of the field-theoretic action non-Gaussian. As a result, the field-based partition function cannot be evaluated exactly. A popular approximation to tackle this difficulty is the self-consistent field approximation, which takes the saddle-point value of the field-theoretic action *L*. However, as alluded to earlier, this approximation does not capture the reaction field effect, which is important to the physics of a polar liquid (*12*, *13*). In our earlier work, we introduced a variational approach to provide an approximate treatment to the partition function, allowing the reaction field effects to be captured (*12*). In this work, we extend the treatment to polar liquid mixtures. The variational approach is carried out by first introducing a Gaussian reference action *L*_0_, so that we can obtain an approximation for the grand potential *W* through an upper bound given by the Gibbs-Feynman-Bogoliubov inequality (*34*)

$$\beta W\leq -\text{ln}\;\Xi_{0}+{\left\langle L-L_{0}\right\rangle }_{0}$$ (17)

where the right-hand side of Eq. 17 is an upper bound of β*W*. Ξ_0_ is the reference partition function given by

$$\Xi_{0}=\int DP\int DG\;e^{-L_{0}[P,G]}$$ (18)

and ⟨𝒪⟩_0_ is the average of an observable 𝒪 evaluated in the reference ensemble, i.e.,

$${\left\langle O\right\rangle }_{0}=\frac{1}{\Xi_{0}}\int DP\int DG\;O[P,G]e^{-L_{0}[P,G]}$$ (19)

For simplicity in notation, we regard the right-hand side of Eq. 17 as the working expression for the grand potential and replace the ≤ sign by = sign.

The reference action must be sufficiently simple so that Eq. 17 can be evaluated. Ideally, it should also be as close to the original action as possible. Thus, we choose a reference action *L*_0_ that keeps the first three terms in *L* but replaces the last nonlinear term by a quadratic functional in the fluctuating field *i***G** that corresponds to a Gaussian with average **F** and variance **A**(**r**)

$$\begin{array}{cc} L_{0}[P,G]= & \frac{1}{2}\int dr\int dr\prime P(r)T(r-r\prime )P(r\prime )\\ & +i\int P(r)\cdot G(r)-\int P(r)\cdot E_{0}(r)\\ & -\frac{1}{2}\int dr\int dr\prime [iG(r)-F(r)]A^{-1}(r-r\prime )[iG(r\prime )-F(r\prime )] \end{array}$$ (20)

**A**(**r**) and **F** are the variational parameters to be determined. The operator **A** describes an effective interaction between the fluctuating fields. Its inverse **A**^−1^ satisfies the relation $\int dr\prime A(r-r\prime )A^{-1}(r\prime -r^{\prime \prime })=1\delta (r-r^{\prime \prime })$. Although it is possible to account for the anisotropy in **A**, we take it to be isotropic since we are concerned with the linear response (i.e., weak external field) limit (*12*), i.e., we write A(r)=a(r)1, where *a*(**r**) is a scalar variational parameter.

Using the reference action ansatz Eq. 20, the right-hand side of Eq. 17 can be evaluated using a series of Gaussian functional integrals. The variational parameters $\tilde{a}(k)$ and $\tilde{F}(k)$ are then determined by setting $\delta W/\delta \tilde{a}(k)=0$ and $\delta W/\delta \tilde{F}(k)=0$. To simplify the results further, we take the limit ${\tilde{h}}_{S}(k)=1$ (i.e., the point dipole limit) whenever this procedure does not produce divergences (*30*). Since we are only interested in the linear response regime, we compute the free energy to the second order in the applied field. At this order, we obtain for $\tilde{a}(k)$

$$\frac{\beta }{\varepsilon_{0}\tilde{a}(k)}=\sum_{S}\frac{\beta {\bar{\mu }}_{S}^{2}\rho_{S}}{3\varepsilon_{0}}$$ (21)

where ρ*_S_* is the number density of the species *S* in the mixture. The dimensionless combination $\beta /(\varepsilon_{0}\tilde{a}(k))$ characterizes the strength of the dipolar interaction in the mixture and has no **k** dependence. For simplicity, we define $y=\beta /(\varepsilon_{0}\tilde{a})$.

The other variational parameter, $\tilde{F}(k)$, is given by the following expression in the linear response regime

$$\tilde{F}(k)=\tilde{K}(k){\tilde{E}}_{0}(k)$$ (22)

where

$$\tilde{K}(k)=\{\begin{array}{cc} -y(1-\frac{\varepsilon_{0}}{\beta }\tilde{T}(k)) & \text{for}\;k\neq 0\\ -(\frac{2y}{y+3})1 & \text{for}\;k=0 \end{array}$$ (23)

The detailed steps in the derivation are presented in the Supplementary Materials.

### The dielectric constant of a mixture

To compute the dielectric constant of a mixture, we consider the variation in the polarization with the applied field. The polarization can be obtained by taking the derivative of the grand potential with respect to the applied field, i.e., **P**(**r**) = − δ*W*/δ**E**_0_(**r**). This allows us to extract the electric susceptibility **χ**_0_, which relates the polarization of the mixture to the applied electric field through $\tilde{P}(k)=\varepsilon_{0}{\tilde{\chi }}_{0}(k){\tilde{E}}_{0}(k)$. Then, we obtain Eq. 8 using the relation $\left(\varepsilon -1)(2\varepsilon +1)/\varepsilon =\text{tr}{\tilde{\chi }}_{0}(k=0\right)$ (*35*). We present the details of the derivation in the Supplementary Materials.

### The free energy of mixing

To derive the free energy of mixing, we assume no change in volume upon mixing and predict the miscibility on the basis of pure-liquid parameters *y_S_* and *v_S_*. The Gibbs free energy of mixing then equals the Helmholtz free energy of mixing. We first perform a Legendre transform on the grand potential to obtain the Helmholtz free energy, *F*, of the mixture. Then, the free energy of mixing, Δ*F*_mix_, is the difference between the free energy of the mixture and the sum of the free energies of the individual unmixed components. The resulting expression for Δ*F*_mix_ is given in Eq. 9. We present the details of the derivation in the Supplementary Materials.

## SUPPLEMENTARY MATERIALS

Supplementary material for this article is available at http://advances.sciencemag.org/cgi/content/full/7/7/eabe7275/DC1
